# Regulation of the Low-Density Lipoprotein Receptor-Related Protein LRP6 and Its Association With Disease: Wnt/β-Catenin Signaling and Beyond

**DOI:** 10.3389/fcell.2021.714330

**Published:** 2021-09-13

**Authors:** Wonyoung Jeong, Eek-hoon Jho

**Affiliations:** Department of Life Science, University of Seoul, Seoul, South Korea

**Keywords:** LRP6, Wnt, cancer, metabolism, signaling

## Abstract

Wnt signaling plays crucial roles in development and tissue homeostasis, and its dysregulation leads to various diseases, notably cancer. Wnt/β-catenin signaling is initiated when the glycoprotein Wnt binds to and forms a ternary complex with the Frizzled and low-density lipoprotein receptor-related protein 5/6 (LRP5/6). Despite being identified as a Wnt co-receptor over 20 years ago, the molecular mechanisms governing how LRP6 senses Wnt and transduces downstream signaling cascades are still being deciphered. Due to its role as one of the main Wnt signaling components, the dysregulation or mutation of LRP6 is implicated in several diseases such as cancer, neurodegeneration, metabolic syndrome and skeletal disease. Herein, we will review how LRP6 is activated by Wnt stimulation and explore the various regulatory mechanisms involved. The participation of LRP6 in other signaling pathways will also be discussed. Finally, the relationship between LRP6 dysregulation and disease will be examined in detail.

## Introduction

**FIGURE 1:**
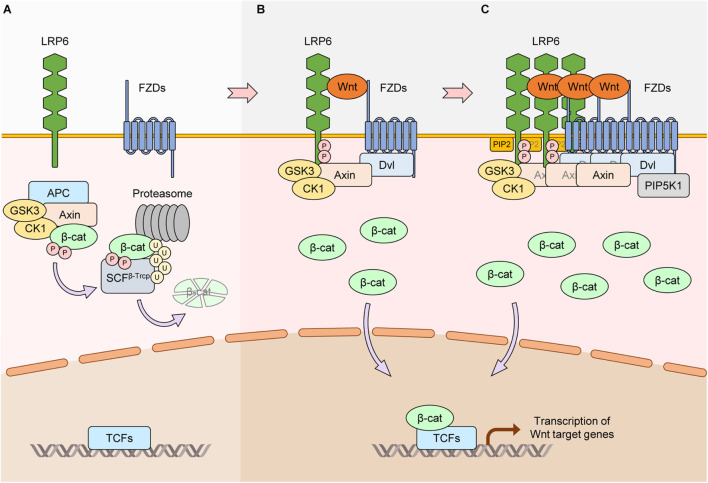
Core activation mechanisms of LRP6 via Wnt stimulation. **(A)** In the absence of Wnt, the scaffold protein Axin together with APC, GSK3, and CK1 form the β-catenin destruction complex. β-catenin interacts with the complex and is phosphorylated by GSK3 and CK1α (CK1). Phosphorylated β-catenin is ubiquitinated by SCF^β–Trcp^ and degraded by the proteasome. Protein levels of β-catenin thus remain low, and β-catenin-dependent transcription of Wnt/β-catenin target genes is suppressed. **(B)** Wnt interacts with the FZD and LRP6 receptors. Axin-bound GSK3 and CK1γ phosphorylate PPPS/TP motifs in the intracellular domain of LRP6. Phosphorylated LRP6 serves as a docking site for Axin, facilitating the interaction between Axin and LRP6 and inhibiting the kinase activity of GSK3. This causes the dissociation and inactivation of the β-catenin destruction complex, leading to β-catenin stabilization and activation of Wnt/β-catenin target gene transcription. **(C)** Treatment of Wnt induces LRP6 aggregates in a DVL-dependent manner. In this condition, FZDs, Axin, and GSK3 can also aggregate with LRP6, generating LRP6 signalosomes. PIP_2_ is generated via DVL-bound PIP5K1. PIP_2_ accelerates the formation of LRP6 signalosomes and phosphorylation of LRP6, resulting in further activation of Wnt/β-catenin signaling.

Wnt signaling has crucial roles in development and tissue homeostasis (Nusse and Clevers, 2017). The interaction between Wnt, Frizzled, and lipoprotein receptor-related protein 5/6 (LRP5/6) activates Wnt signaling. If the main output of Wnt signaling activation is stabilization of the transcriptional activator β-catenin, the pathway is known as canonical Wnt or Wnt/β-catenin signaling (hereafter referred to as “Wnt/β-catenin signaling”). In the absence of Wnt, the scaffold protein Axin together with adenomatous polyposis coli (APC), glycogen synthase kinase 3β (GSK3β), and casein kinase 1 alpha (CK1α) form the so called destruction complex that binds cytoplasmic β-catenin, leading to its phosphorylation by CK1α and GSK3β. Phosphorylated β-catenin is ubiquitinated by the SCF^β–Trcp^ E3 ubiquitin ligase complex, a process that targets it for proteasomal degradation (Aberle et al., 1997; Kitagawa et al., 1999; Liu et al., 2002). In the presence of Wnt, the β-catenin destruction complex is recruited to the plasma membrane and inactivated (Figure 1A). As a result, β-catenin is stabilized and then translocates to the nucleus to activate the expression of target genes involved in cell proliferation, differentiation, stem cell self-renewal and many other biological processes (MacDonald et al., 2009). In non-canonical Wnt signaling, Wnt (e.g., Wnt5a) transduces signaling without β-catenin stabilization by activating alternative downstream cascades such as JUN kinase, planar cell polarity (PCP), or calcium signaling (Kikuchi et al., 2009). It is well known that dysregulation of Wnt signaling causes developmental disorders and several diseases such as cancer (Nusse and Clevers, 2017). Notably, hyper-activation of β-catenin, due to mutations in *APC*, *AXIN*, or *CTNNB1* (gene for β-catenin), is a well-known risk factor for carcinogenesis, especially colon cancer (Bugter et al., 2021).

The type I single transmembrane protein LRP6 is a member of the LDLR gene family of receptors that is highly conserved among species (Pinson et al., 2000; Tamai et al., 2000; Wehrli et al., 2000). The extracellular region of LRP6 contains four YWTD (Tyr-Trp-Thr-Asp)-type β-propellers, followed by EGF-like domains (E1–E4), and three LDLR type A domains (Cheng et al., 2011), and its intracellular region contains five PPPS/TP (Pro-Pro-Pro-Ser/Thr-Pro) motifs (Tamai et al., 2004). Formation of the Wnt-FZD-LRP6 ternary complex at the plasma membrane (i.e., Wnt-on state) induces phosphorylation of the intracellular region of LRP6 (MacDonald and He, 2012). Phosphorylation of LRP6 is therefore considered a hallmark of Wnt/β-catenin signaling activation. Contrary to Wnt, the secreted Wnt modulator Dickkopf (Dkk) binds to LRP6 and promotes its membrane clearance, thereby functioning as an LRP6 antagonist (Mao B. et al., 2001; Mao et al., 2002). Owing to its importance in Wnt/β-catenin signaling transduction, mutation or dysregulation of LRP6 is implicated in several diseases (Joiner et al., 2013). LRP5, which is a paralog of LRP6 and shares 71% sequence conservation (Tamai et al., 2000), plays a similar role as LRP6 in transducing Wnt/β-catenin signaling (Mao J. et al., 2001); however, the biological functions of LRP6 and LRP5 differ significantly (Joiner et al., 2013). In this review we will mainly focus on LRP6. We will describe the molecular mechanisms governing Wnt-dependent LRP6 activation, and discuss how LRP6 function is regulated by various factors. We will also discuss LRP6’s role in the regulation of downstream Wnt/β-catenin signaling, provide examples of its involvement in Wnt/β-catenin-independent pathways, and illustrate how dysregulation or mutation of LRP6 can lead to several diseases.

## Core Mechanisms of LRP6 Activation via Wnt Stimulation

In 2000, LRP6 was identified as a co-receptor for Wnt and FZD to transduce Wnt/β-catenin signaling (Pinson et al., 2000; Tamai et al., 2000; Wehrli et al., 2000). The extracellular domain of LRP6 interacts with Wnt and activates Wnt/β-catenin signaling at the plasma membrane. LRP6 with a truncated extracellular domain is constitutively active and can potentiate Wnt/β-catenin signaling independently of Wnt (Liu et al., 2003). Conversely, LRP6 with a truncated intracellular domain acts as a dominant-negative form, inhibiting Wnt/β-catenin signaling (Tamai et al., 2000). There are five PPPS/TP motifs in the LRP6 intracellular domain, and the serine/threonine residues in these motifs are phosphorylated upon Wnt stimulation (Tamai et al., 2004). GSK3β and CK1γ are the main kinases that phosphorylate the PPPS/TP motifs and their flanking regions, respectively (Davidson et al., 2005; Zeng et al., 2005). Dishevelled (DVL) proteins are essential for Wnt-induced LRP6 aggregation with FZD, and the complex formed between LRP6, FZD, and DVL relies on the DIX and PDZ domains of DVL (Zeng et al., 2008; Figure 1B). In the Wnt-on state, additional Wnt/β-catenin signaling components such as Axin, CK1α, and GSK3β form a complex with LRP6 known as the signalosome (Bilic et al., 2007). Signalosome formation leads to further LRP6 phosphorylation by GSK3β that in turn promotes more aggregation of Wnt/β-catenin signaling components (Zeng et al., 2008). The end result is increased dissociation of β-catenin away from the destruction complex, allowing it to accumulate in the cytoplasm and then translocate to the nucleus (Cselenyi et al., 2008; Wu et al., 2009). Wnt3a-induced activation of LRP6 is rapid, and aggregation of components involved in Wnt/β-catenin signaling can be observed as early as 30 min by live cell imaging (Bilic et al., 2007). Another important player in the signalosome is PIP5K1, a phosphatidylinositol phosphate kinase whose activation is mediated by FZD and DVL (Pan et al., 2008). Activation of PIP5K1 leads to production of phosphatidylinositol 4,5-bisphosphate (ptdIns(4,5)P_2_), which in turn induces aggregation and phosphorylation of LRP6 (Pan et al., 2008; Figure 1C). Hence, non-protein components such as phospholipids can also play crucial roles in LRP6-mediated Wnt/β-catenin signaling.

## Regulation of LRP6 Function and Downstream Signaling

### Phosphorylation

**FIGURE 2:**
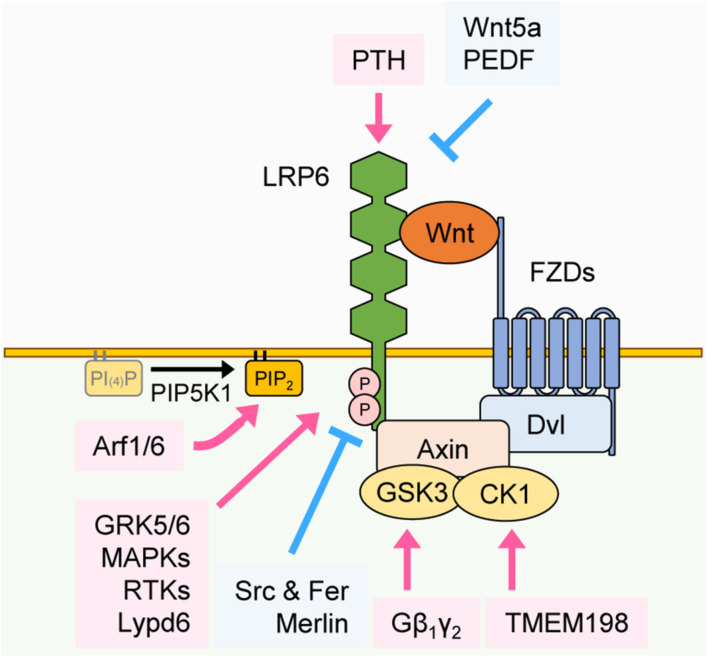
Regulation of LRP6 phosphorylation. Phosphorylation of LRP6 can be regulated by various proteins through distinct mechanisms. PTH interacts with the extracellular domain of LRP6 and facilitates its phosphorylation. In contrast, Wnt5a and PEDF interact with the extracellular domain of LRP6 and inhibit LRP6 phosphorylation. GRK5/6, MAPKs, and RTKs facilitate phosphorylation of LRP6 in the cytoplasm, and Lypd6 facilitates phosphorylation of LRP6 in the plasma membrane. Src and Fer and Merlin inhibit phosphorylation of LRP6 in the cytoplasm. Gβ_1_γ_2_ and TMEM198 promote phosphorylation of LRP6 in GSK3 and CK1-dependent manners, respectively. Arf1/6 facilitates the phosphorylation of LRP6 in a PIP_2_-dependent manner.

As described above, in the presence of Wnt, the five PPPS/TP motifs in the intracellular domain of LRP6 are mainly phosphorylated by GSK3β and CK1γ. However, additional ligands, kinases or interacting proteins have also been shown to regulate LRP6 phosphorylation and thus influence Wnt/β-catenin signaling. First, we review how these components affect LRP6 phosphorylation and positively regulate Wnt/β-catenin signaling. Similar to GSK3β, G protein-coupled receptor kinases 5 and 6 (GRK5/6), mitogen-activated protein kinases (MAPKs), such as p38, extracellular signal regulated kinase 1 and 2 (ERK1/2), and c-Jun N-terminal kinases 1 (JNK1) also interact with LRP6 and induce phosphorylation of its PPPS/TP motifs (Chen et al., 2009; Červenka et al., 2011; Figure 2). For example, fibroblast growth factor 2 (FGF2)-induced ERK activation leads to phosphorylation of the S1490 or T1572 residues of LRP6, resulting in Wnt/β-catenin signaling activation (Krejci et al., 2012). Parathyroid hormone (PTH), a crucial factor for bone formation, acts as another LRP6 regulator by forming a ternary complex with PTH1 receptor (PTH1R) and LRP6 to facilitate PPPS/TP phosphorylation (Wan et al., 2008). In addition, several proteins interact with LRP6 and thereby enhance its phosphorylation and Wnt/β-catenin signaling by modulating LRP6 localization or acting as a scaffold for LRP6 and other Wnt components. For instance, the G protein Gβ_1_γ_2_ promotes GSK3 localization to the plasma membrane, which in turn promotes LRP6 phosphorylation (Jernigan et al., 2010). DVL is well-known for playing a crucial role in signalosome formation. Ectopic expression of the DVL DIX domain fused to the LRP6 intracellular domain promotes Wnt/β-catenin signaling via formation of cytoplasmic signalosomes and induction of LRP6 phosphorylation, suggesting that stable LRP6-DVL interactions are essential for the maintenance of LRP6 phosphorylation (Metcalfe et al., 2010). Interestingly, transmembrane protein 198 (TMEM198), a previously uncharacterized seven-transmembrane protein, acts as a scaffold protein for CK1γ and LRP6 (Liang et al., 2011) and thus enhances phosphorylation of LRP6 (Figure 2).

Changes in plasma membrane lipid composition can also affect the phosphorylation of LRP6 and subsequent Wnt/β-catenin signaling activation. APC membrane recruitment protein 1 (Amer1) translocates to the plasma membrane in a PtdIns(4,5)P_2_-dependent manner, where it recruits Axin, CK1γ, and GSK3β to promote LRP6 phosphorylation (Tanneberger et al., 2011). ADP-ribosylation factors 1 and 6 (Arf1/6) switch to the GTP-bound active form upon Wnt3a treatment, which facilitates the production of PtdIns(4,5)P_2_ (PIP_2_) and subsequent LRP6 phosphorylation (Kim W. et al., 2013). LY6/PLAUR domain-containing 6 protein (Lypd6) interacts with LRP6 and induces its localization to lipid rafts (Özhan et al., 2013). A lipid raft is a specific region in the plasma membrane where lipid components such as sphingolipid and cholesterol are enriched and cellular signaling is activated (Sezgin et al., 2017). Therefore Lypd6 potentiates LRP6 phosphorylation and activates Wnt/β-catenin signaling (Figure 2).

Next, we review other proteins that influence LRP6 phosphorylation and negatively regulate Wnt/β-catenin signaling. In contrast to CK1γ, whose phosphorylation of LRP6 enhances Wnt/β-catenin signaling, CK1ε inhibits Wnt/β-catenin signaling by interacting with and phosphorylating LRP6 at the S1420 and S1430 residues that are not present in PPPS/TP motifs (Swiatek et al., 2006; Figure 2). Moreover, Src and Fer tyrosine kinases phosphorylate LRP6 tyrosine residues near the PPPS/TP motifs, which leads to reduction of LRP6 cell surface levels and blockage of signalosome formation (Chen et al., 2014). It has been shown that several ligands for LRP6 inhibit its phosphorylation and suppress Wnt/β-catenin signaling. Wnt5a is mainly involved in non-canonical Wnt signaling. However, by recruiting Wnt receptors away from canonical Wnts (e.g., Wnt3a), Wnt5a can inhibit the phosphorylation of LRP6 and therefore act as a negative regulator of Wnt/β-catenin signaling (Grumolato et al., 2010; Sato et al., 2010). Pigment epithelium-derived factor (PEDF) interacts with the extracellular domain of LRP6, inhibiting LRP6-FZD interaction and phosphorylation of LRP6 (Park et al., 2011). Protein interactions in the intracellular region of LRP6 also mediate the inhibition of LRP6 phosphorylation. Merlin, a main player in the Hippo signaling pathway, interacts with LRP6 and inhibits its phosphorylation (Kim et al., 2016). Merlin-induced inhibition of LRP6 phosphorylation is counteracted by Wnt3a treatment, which, by inducing phosphorylation of merlin through P21 activated kinase 1 (PAK1), promotes merlin dissociation from LRP6 (Kim et al., 2016; Figure 2). Overall, these studies suggest that several proteins, by acting as kinases, ligands, or binding partners for LRP6, are crucial for regulating LRP6 phosphorylation and Wnt/β-catenin signaling, either in a positive or negative manner.

### Internalization

**FIGURE 3:**
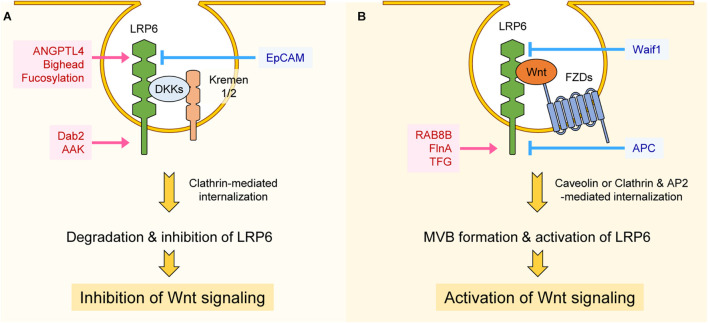
Regulation of LRP6 internalization. **(A)** Interaction between LRP6, DKKs, and Kremen1/2 promotes clathrin-mediated internalization, resulting in degradation or inhibition of LRP6. ANGPTL4, Bighead, and fucosylation of LRP6 promotes internalization of LRP6 at the extracellular level, thereby inhibiting LRP6 function and Wnt/β-catenin signaling. Dab2 and AAK function at the intracellular level and promote clathrin-mediated internalization of LRP6, resulting in Wnt/β-catenin signaling suppression. On the other hand, EpCAM inhibits LRP6-DKK-Kremen1/2 complex formation, resulting in Wnt signaling activation. **(B)** Interaction among LRP6, FZDs, and Wnt promotes caveolin-mediated internalization, resulting in LRP6 activation and MVB formation. RAB8B, FinA, and TFG promote Wnt3a-mediated internalization of LRP6 and Wnt/β-catenin signaling at the intracellular level. However, Waif1 compromises Wnt-LRP6 interaction and inhibits Wnt/β-catenin signaling. APC inhibits Clathrin and AP2-mediated internalization of LRP6 at the intracellular level, resulting in suppression of Wnt/β-catenin signaling.

Receptor-mediated internalization plays a crucial role in signal transduction. LRP6 is internalized after binding to ligands, and internalized LRP6 can either positively or negatively regulate Wnt/β-catenin signaling. For instance, the secreted Wnt modulator Dkk1, by forming a ternary complex with the single transmembrane protein Kremen1/2 and LRP6, internalizes LRP6 and decreases its plasma membrane levels, leading to Wnt/β-catenin signaling inhibition (Bafico et al., 2001; Mao B. et al., 2001; Semënov et al., 2001; Mao et al., 2002; Figure 3A). Internalization of LRP6 via Dkk1 also leads to decreased LRP6 phosphorylation by CK1γ (Sakane et al., 2010). Angiopoietin-like 4 protein (ANGPTL4) is another secretory protein that, by forming a complex with syndecan and LRP6, induces LRP6 internalization and decreases its surface levels (Kirsch et al., 2017). Similar to ANGPTL4, the secretory protein Bighead interacts with LRP6 and promotes its endocytosis and lysosomal degradation, resulting in suppression of Wnt/β-catenin signaling (Ding et al., 2018). Glycosylation of LRP6 can also influence its internalization. LRP6 can be fucosylated, a process that promotes the internalization of lipid raft-localized LRP6. This process prevents formation of the Wnt-FZD-LRP6 complex and thus inhibits Wnt/β-catenin signaling (Hong et al., 2020; Figure 3A). Wnt-activated inhibitory factor 1 protein (Waif1), a transmembrane protein, interacts with LRP6 and inhibits Wnt3a-induced LRP6 internalization into endocytic vesicles, thereby reducing Wnt/β-catenin signaling (Kagermeier-Schenk et al., 2011; Figure 3B).

Clathrin, a protein with a prominent role in cellular vesicle formation, promotes Dkk-mediated LRP6 internalization and thus acts as a negative regulator Wnt/β-catenin signaling (Yamamoto et al., 2008). Interestingly, clathrin can also promote LRP6 internalization in the presence of Wnt. This is because Wnt3a treatment, by inducing S1579 phosphorylation of LRP6, enhances LRP6 binding to disabled-2 (Dab2), an interaction that promotes clathrin-mediated LRP6 internalization (Jiang et al., 2012). Similar to Dab2, long-term treatment of Wnt3a (6–8 h) induces phosphorylation of adaptor related protein complex 2 subunit mu 1 (AP2M1) through AP2-associated kinase 1 (AAK1), and phosphorylated AP2M1 activates clathrin-mediated LRP6 internalization, once again leading to negative regulation of Wnt/β-catenin signaling (Agajanian et al., 2019). Therefore Dab2 and AAK1 seem to alleviate hyper-activation of Wnt/β-catenin signaling induced by Wnt stimulation (Figure 3A). Whereas clathrin is known for having a role in internalization of LRP6 and inhibition of Wnt/β-catenin signaling, it is reported that clathrin and AP2 act as components of the LRP6 signalosome, being recruited to the signalosome in a PtdIns(4,5)P_2_-dependent manner (Kim I. et al., 2013). In this context, clathrin and AP2 seem to play a role in cell surface signalosome formation for activation of Wnt/β-catenin signaling, as well as in LRP6 internalization. Interestingly, it is reported that APC, a main component of the β-catenin destruction complex, is also involved in LRP6 internalization. APC directly interacts with clathrin and AP2 to inhibit clathrin-induced LRP6 internalization, a process that generally leads to constitutive ligand-independent Wnt/β-catenin activation (Saito-Diaz et al., 2018). APC thus blocks Wnt/β-catenin signaling in two different contexts: in the cytoplasm, by forming destruction complex, and in the plasma membrane, by preventing LRP6 internalization (Saito-Diaz et al., 2018; Figure 3B).

It is well known that several components positively regulate Wnt/β-catenin signaling by modulating internalization of LRP6. Epithelial-cell-adhesion molecule (EpCAM) interacts with Kremen1 and inhibits Kremen1-Dkk2-LRP6 complex formation and internalization, resulting in activation of Wnt/β-catenin signaling (Lu et al., 2013; Figure 3A). In the presence of Wnt, LRP6 together with FZD, Axin, and GSK3β are internalized in caveolin-enriched vesicles (Yamamoto et al., 2006). GSK3β is sequestered in complex with LRP6 in multivesicular bodies (MVBs) and vastly reduces its phosphorylation of β-catenin, leading to activation of Wnt/β-catenin signaling (Taelman et al., 2010). There are many components involved in the activation of Wnt/β-catenin signaling via internalization of LRP6. These include Rab GTPase RAB8B protein and actin-binding protein filamin A (FlnA), both of which promote caveolin-mediated LRP6 internalization (Demir et al., 2013; Lian et al., 2016). Upon Wnt3a treatment, RAB8B interacts with LRP6 and is recruited to the signalosome complex, where it enhances caveolin-mediated internalization of LRP6 and subsequent Wnt/β-catenin signaling activation (Demir et al., 2013). FlnA interacts with LRP6, and loss of FlnA impairs internalization of LRP6 and activation of Wnt/β-catenin signaling, resulting in decreased proliferation of neural progenitor cells (Lian et al., 2016; Figure 3B). Recently, LRP6 proximity proteins induced upon short-term Wnt3a treatment were identified using an LRP6-Apex2 fusion protein (Colozza et al., 2020). Among them, Trk fused gene protein (TFG) appears to have an important role in Wnt3a-mediated LRP6 internalization and activation of Wnt/β-catenin signaling (Colozza et al., 2020; Figure 3B).

### Regulation of LRP6 Maturation and Stability

**FIGURE 4:**
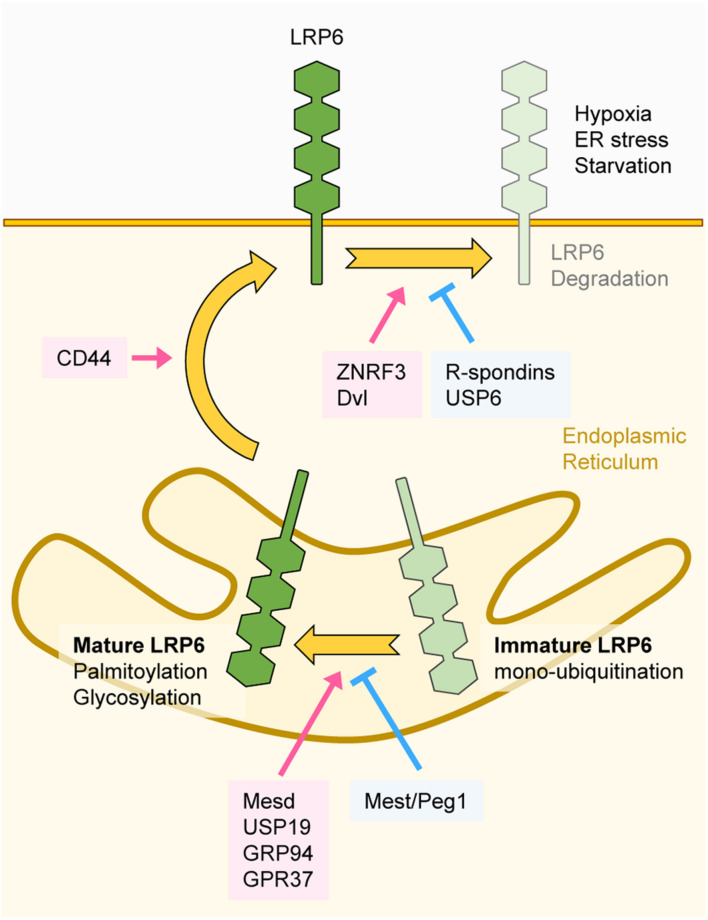
Regulation of LRP6 maturation and stability. The transmembrane protein LRP6 undergoes several folding and maturation processes to translocate to the plasma membrane and properly function as a Wnt co-receptor. Mesd, USP19, GRP94, and GPR37 promote proper folding of LRP6. Glycosylation and palmitoylation of LRP6 are essential for its maturation. Mest/Peg1 inhibits glycosylation of LRP6 and immature LRP6 is mono-ubiquitinated, resulting in ER retention. Folded and mature LRP6 localizes to the plasma membrane, and CD44 promotes membrane localization of LRP6. ZNRF3 and DVL downregulate LRP6 protein levels, and USP6 and R-spondins antagonize ZNRF3 function. Extracellular stimuli such as hypoxia, ER stress, and starvation also degrade LRP6.

Proper folding and maturation are essential for LRP6 to carry out its functions at the plasma membrane, and there are several components involved in these processes. Although mature LRP6 is known to be a stable protein (Perrody et al., 2016), its stability can be altered by extracellular stimuli or regulatory factors. Mesoderm development LRP chaperone protein (Mesd) localizes to the endoplasmic reticulum (ER), where it enhances the maturation and plasma membrane localization of LRP6 (Hsieh et al., 2003; Figure 4). Several proteins are involved in Mesd-mediated maturation of LRP6. The ER heat shock protein Grp94 promotes interaction between LRP6 and Mesd, and Grp94-deficiency suppresses LRP6 maturation (Liu et al., 2013). The transmembrane glycoprotein CD44 interacts with LRP6 and promotes Mesd-mediated membrane localization of LRP6 (Schmitt et al., 2015). The Parkinson’s disease-associated receptor (GPR37) acts as an additional chaperone for LRP6 and promotes the maturation and membrane localization of LRP6. Additionally, GPR37 also inhibits ER-associated degradation of LRP6 and thereby enhances the protein levels of LRP6 (Berger et al., 2017).

Post-translational modifications (PTMs) have also been found to be important for regulating LRP6 folding and maturation. By using an endogenous antibody against LRP6, it has been found that LRP6 is N-glycosylated, and that N-glycosylation is necessary for the membrane localization of LRP6 (Khan et al., 2007). On the other hand, mesoderm-specific transcript/paternally expressed gene 1 (Mest/Peg1) represses glycosylation and plasma membrane localization of LRP6 (Jung et al., 2011), resulting in repression of Wnt/β-catenin signaling. Moreover, palmitoylation on a juxtamembrane cysteine of LRP6 enables its translocation from the ER to the plasma membrane (Abrami et al., 2008; Figure 4). If this process is hindered, mono-ubiquitination on the K1403 residue of LRP6 is promoted, leading to ER retention (Abrami et al., 2008). Further studies revealed that LRP6 mono-ubiquitination can be negatively regulated by the deubiquitinase USP19. Deubiquitination of LRP6 by USP19 facilitates LRP6 translocation to the plasma membrane through proper folding and palmitoylation (Perrody et al., 2016).

The R-spondin family members are secreted proteins that influence LRP6 stability (Wei et al., 2007). R-spondins are high affinity ligands for the Leucine-rich repeat-containing G-protein coupled receptors 4/5 (LGR4/5) and the transmembrane E3 ubiquitin ligases ZNRF3/RNF43 (Carmon et al., 2011; Hao et al., 2012; Koo et al., 2012). In the absence of R-spondins, ZNRF3/RNF43 ubiquitinate Wnt receptors and promote their clearance from the plasma membrane. Binding of R-spondins to LGR4/5 and ZNRF3/RNF43 induces ZNRF3/RNF43 internalization, leading to Wnt receptor stabilization. R-spondins thus regulate the activity and phosphorylation of LRP6 by stabilizing it at the plasma membrane (Carmon et al., 2011; Hao et al., 2012; Koo et al., 2012). Further studies revealed that DVL recruits ZNRF3 to the plasma membrane and mediates ZNRF3-dependent downregulation of LRP6 (Jiang et al., 2015). Consistently, upregulation LRP6 protein levels was observed in DVL1/2/3 knockout cells, owing to lack of LRP6 plasma membrane clearance by ZNRF3 (Jiang et al., 2015). Therefore, DVL seems to have dual role in the regulation of Wnt/β-catenin signaling since it promotes both aggregation and destabilization of LRP6 at the plasma membrane. Contrary to ZNRF3, the deubiquitinase USP6 increases LRP6 membrane levels and potentiates Wnt/β-catenin signaling by antagonizing the function of ZNRF3 (Madan et al., 2016).

Cellular stress can influence LRP6 stability. Chemically induced ER stress or hypoxia reduces the stability of LRP6, resulting in inhibition of Wnt/β-catenin signaling (Xia et al., 2019). Moreover, *O*-GlcNAcylation, a PTM that induces the attachment of *N*-acetylglucosamine (GlcNAc) to Ser/Thr residues, also plays a crucial role in LRP6 stability. During serum starvation, *O*-GlcNAcylation of LRP6 is reduced, which is followed by lysosomal degradation of LRP6 (Jeong et al., 2020; Figure 4).

## β-Catenin-Independent Signaling Via Activation of LRP6

It is generally assumed that the primary output of LRP6 activity is directly associated with alterations in Wnt/β-catenin signaling. However, several studies have revealed that LRP6 affects not only Wnt/β-catenin signaling, but other signaling pathways as well. These include non-canonical Wnt signaling, Wnt-dependent stabilization of proteins (Wnt/STOP) signaling, G protein-coupled receptor (GPCR) and Hippo signaling.

**FIGURE 5:**
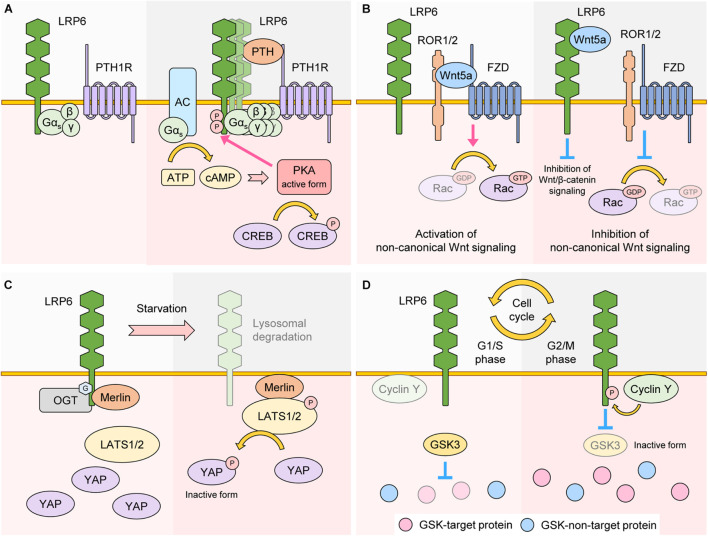
Role of LRP6 as a regulator of other signaling. **(A)** LRP6 as a regulator of GPCR signaling. In the basal state, LRP6 interacts with G protein Gα_s_. In the presence of the GPCR ligand PTH, a LRP6-PTH-PTH1R ternary complex is formed, which promotes aggregation of LRP6 and membrane localization of Gα_s_. Production of cAMP is also upregulated in a Gα_s_-AC-dependent manner. cAMP activates PKA, which promotes phosphorylation of LRP6 and CREB, two well-known downstream targets of PKA. **(B)** LRP6 as a regulator of non-canonical Wnt signaling. Wnt5a interacts with ROR1/2 and FZD, resulting in activation of Rac, a non-canonical Wnt signaling target. Wnt5a can also interact with LRP6. In these conditions, the binding affinity of ROR1/2 and FZD to Wnt5a is reduced. As a result, Rac becomes inactive and non-canonical Wnt signaling is inhibited. Because LRP6-Wnt5a binding weakens LRP6-Wnt3a interaction, Wnt/β-catenin signaling is also inhibited. **(C)** LRP6 as a regulator of Hippo signaling. In a nutrient rich state, LRP6 is *O*-GlcNAcylated and interacts with Merlin. In this condition, activity of LATS1/2 is maintained at low levels, resulting in stabilization and activation of YAP. In nutrient starvation conditions, *O*-GlcNAcylation and protein levels of LRP6 are both downregulated, and Merlin changes its binding partner from LRP6 to LATS1/2, resulting in activation of LATS1/2. YAP is phosphorylated by LATS1/2 and becomes inactive. **(D)** LRP6 as a regulator of Wnt/STOP signaling. In G1/S phase, cyclin Y protein levels are less abundant and the phosphorylation state of LRP6 is low, resulting in higher GSK3 activity. GSK3-target proteins are thus phosphorylated and targeted for proteasomal degradation. In G2/M phase, cyclin Y protein levels peak and promote LRP6 phosphorylation, resulting in inactivation of GSK3 and stabilization of GSK3-target proteins.

The interaction of GPCR ligands with their associated receptors initiates GPCR signaling via activation of the G protein Gα, which mediates the activity of downstream effector proteins. LRP6 interacts with and promotes membrane localization of the G protein Gα_s_ (Wan et al., 2011). Moreover, in the presence of GPCR ligands such as PTH, LRP6 stimulates the production of cyclic AMP (cAMP) via Gα_s_, and newly generated cAMP activates protein kinase a (PKA). Previous reports have shown that PTH facilitates LRP6 phosphorylation and activation of Wnt/β-catenin signaling in osteoblasts (Wan et al., 2008), suggesting that LRP6 is involved in both, Wnt/β-catenin and GPCR signaling, in the context of bone formation (Wan et al., 2011; Figure 5A).

The extracellular region of LRP6 interacts with Wnt5a, and this interaction inhibits activation of Rac1, a target protein of non-canonical Wnt signaling (Bryja et al., 2009). In addition, Wnt5a treatment interferes with the interaction between Wnt3a and LRP6, resulting in not only inhibition of Wnt/β-catenin signaling, but activation of non-canonical Wnt signaling as well (Bryja et al., 2009; Grumolato et al., 2010; Figure 5B).

Hippo signaling is a crucial regulator of organ size and cellular homeostasis (Pan, 2010). Activation of Hippo signaling leads to serial phosphorylation and activation of STE20-like serine/threonine kinases 1/2 (MST1/2) and Large Tumor Suppressor 1/2 (LATS1/2). Activated LATS1/2 phosphorylates Yes-associated protein (YAP) and WW domain containing transcription regulator protein 1 (TAZ). As a result, phosphorylated YAP and TAZ undergo 14-3-3-mediated cytoplasmic retention or proteasomal degradation, a process which blocks their transcriptional activity and thereby inhibits cell proliferation and survival (Meng et al., 2016). It has been revealed that LRP6 is involved in the regulation of Hippo signaling. YAP/TAZ are incorporated into the β-catenin destruction complex, and treatment with Wnt3a or overexpression of LRP6 stabilizes the protein levels of YAP/TAZ in a similar fashion to β-catenin, leading to increased YAP/TAZ transcriptional activity (Azzolin et al., 2014). Another study revealed that loss of LRP6 via serum starvation promotes the dissociation of Merlin from LRP6, which activates Hippo signaling by facilitating Merlin-LATS interaction. As a result, loss of LRP6 enables phosphorylation of YAP, inhibiting its transcriptional activity (Jeong et al., 2020; Figure 5C).

Recent studies have shown that LRP6 phosphorylation peaks during the G2/M phase of the cell cycle and that this peak is dependent on cyclin Y and its cyclin dependent kinase 14 (CDK14). Originally, identified via kinome-wide RNAi screening in *Drosophila* cells, the cyclin Y-CDK14 complex phosphorylates the PPPS/TP S1490 residue of LRP6 (Davidson et al., 2009). Cyclin Y protein levels peak during G2/M, which explains the cell cycle dependence of LRP6 phosphorylation (Davidson et al., 2009). Mechanistically, G2/M phosphorylation of LRP6 by cyclin Y-CDK14 primes LRP6 for incoming Wnts, which in turn suppresses the activity of GSK3 and prevents GSK3 target proteins from proteasomal degradation (Taelman et al., 2010; Figure 2). Suppression of GSK3 during G2/M thus leads to an overall increase in protein stabilization, ensuring proper cell division and growth (Acebron et al., 2014). Importantly, this new Wnt sub-branch, also known as Wnt/STOP pathway, is completely dependent on LRP6 (Acebron et al., 2014; Acebron and Niehrs, 2016). Another player in the Wnt/STOP pathway is Caprin-2, which acts as a scaffold for LRP6 and cyclin Y and thereby promotes LRP6 phosphorylation during G2/M (Wang et al., 2016). Moreover, B-cell CLL/lymphoma 9 protein (BCL9) is phosphorylated at the T172 residue by cyclin dependent kinase 1 (CDK1), and phosphorylated BCL9 inhibits LRP6 degradation thereby acting as a positive regulator of Wnt/STOP signaling (Chen et al., 2018). These data suggest that LRP6 phosphorylation-mediated Wnt signaling can be transduced in a β-catenin-independent manner (Figure 5D).

## LRP6 Dysregulation and Disease

### Cancer

Dysregulation of Wnt/β-catenin signaling is highly associated with cancer, and mutations in AXIN, APC, and β-catenin often lead to increased cancer formation and metastasis (Bugter et al., 2021). Similarly, dysregulation of LRP6 is also involved in cancer. *LRP6* is highly expressed in several cancer cell lines and overexpression of *LRP6* promotes cancer cell proliferation (Li et al., 2004). More specifically, LRP6 is a well-known regulator of breast cancer: *LRP6* expression is frequently upregulated in breast cancer tissue, and respective overexpression or knockdown of *LRP6* induces or inhibits breast tumorigenesis (Li et al., 2004; Lindvall et al., 2009; Liu et al., 2010; Zhang et al., 2010). The role of LRP6 in breast cancer tumorigenesis is highly dependent on Wnt/β-catenin signaling. If antibodies that block LRP6-Wnt1 or LRP6-Wnt3a interactions are administered in mice, Wnt/β-catenin signaling is blocked and breast tumor growth is suppressed (Ettenberg et al., 2010). In breast cancer tissue, high expression of the Sry-related HMG box 9 protein (SOX9) activates Wnt/β-catenin signaling by inducing *LRP6* expression (Wang et al., 2013). LRP6 also plays a role in breast cancer metastasis. N-myc downstream regulated gene-1 protein (NDRG1) interacts with LRP6 and suppresses LRP6-mediated Wnt signaling activation, resulting in inhibition of breast cancer metastasis (Liu et al., 2012). Contrastingly, in the absence of Wnt3a, LRP6 inhibits FZD8-mediated non-canonical Wnt signaling by interacting with the extracellular domain of FZD8 (Ren et al., 2015). As a result, breast tumor metastasis, which is usually promoted by non-canonical Wnt signaling, is inhibited through the extracellular domain of LRP6 (Ren et al., 2015). Therefore, LRP6 seems to play a dual role in breast tumor metastasis that depends on the presence or absence of Wnt.

Another cancer with which LRP6 is highly correlated is liver cancer. *LRP6* is highly expressed in tumors of liver cancer patients, and overexpression of *LRP6* promotes liver cancer cell proliferation and tumor growth (Tung et al., 2012). Several components are involved in liver cancer progression via regulation of LRP6. For example, expression of stearoyl-CoA desaturase (SCD) is increased in liver tumors, where it promotes the production of monounsaturated fatty acids (MUFA) (Lai et al., 2017). MUFA induces expression of *LRP6* and activation of Wnt/β-catenin signaling, which then activates expression of *SCD*, functioning as a positive feedback loop (Lai et al., 2017). Connective tissue growth factor (CTGF) is highly expressed in liver cancer patients, and CTGF promotes phosphorylation of LRP6 (Jia et al., 2017). Finally, expression of long non-coding RNA DLGAP1-AS1 is increased in liver cancer tissue, where it inhibits miR-26a/b-5p, a negative regulator of *LRP6* expression (Lin et al., 2020).

In colorectal cancer, elevated activity of LRP6 has been reported. LRP6 phosphorylation was also found to be enhanced in colorectal cancer tissue from patients (Lemieux et al., 2015). In colorectal cancer cells, gain of function mutations in KRAS increase LRP6 phosphorylation, resulting in activation of Wnt/β-catenin signaling (Lemieux et al., 2015). CD110 receptor-expressing colorectal cancer tumor-initiating cells (TICs) are activated via thrombopoietin in blood vessels. In TICs, production of acetyl-CoA is promoted via degradation of lysine, and the LRP6 K802 residue is acetylated (Wu et al., 2015). Acetylation of LRP6 leads to its phosphorylation in a CK1γ-dependent manner, leading to activation of Wnt/β-catenin signaling. As a result, self-renewal and metastasis of colorectal cancer TICs are enhanced (Wu et al., 2015). V-set and transmembrane domain containing 2A (VSTM2A) is a secretory protein that is lowly expressed in colorectal cancer tissue (Dong et al., 2019). VSTM2A interacts with the extracellular domain of LRP6 and inhibits LRP6 phosphorylation, thereby inducing its lysosomal degradation, and suppressing colorectal cancer progression (Dong et al., 2019).

In addition to breast, liver, and colorectal cancer, the role of LRP6 in other cancers has been studied. In prostate cancer, high expression levels of caveolin-1 and *LRP6* are detected, and these two proteins activate Wnt/β-catenin signaling and glycolysis through Akt signaling. The end result is increased prostate cancer cell proliferation (Tahir et al., 2013). Through mass spectrometry-based proteomic analyses of mass spectrometry data, it was identified that *LRP6* expression is high in oral squamous cell carcinoma (OSCC) (Yuan et al., 2017). In addition, LRP6 increases the protein levels of fibroblast growth factor 8 (FGF8), which can act as an oncogene and promote OSCC progression (Yuan et al., 2017). LRP6 is also involved in regulating the activity of cancer-associated fibroblasts (CAFs). In the stroma of breast, colon, and ovarian cancers, *Dkk3* expression and internalization with Kremen1/2 are enhanced, resulting in upregulation of LRP6 protein levels (Ferrari et al., 2019). Finally, LRP6 stabilizes not only β-catenin, but also YAP/TAZ. Stabilized YAP/TAZ enters the nucleus where it enhances tumorigenic activity in various cancer types (Ferrari et al., 2019).

### Neurodegeneration

Cognitive and behavioral disorders caused by functional neuron failure and neuronal death are referred to as neurodegeneration. Representative examples include Alzheimer’s, Parkinson’s, and Huntington’s diseases. The causes of neurodegeneration include genetic mutations, protein aggregation, mitochondrial dysfunction, etc. However, the molecular mechanisms underlying neurodegeneration still require further elucidation (Gan et al., 2018). The relationship between Wnt signaling dysregulation and neurodegeneration has been reported, and several studies have shown that mutations in LRP6 are associated with neurodegeneration.

Through genome-wide screening, it was identified that a single nucleotide polymorphism (SNP) in the 1062 residue of LRP6, which converts isoleucine to valine (hereafter referred to as Ile1062Val), leads to reduced Wnt/β-catenin signaling and is implicated in Alzheimer’s disease (De Ferrari et al., 2007). In addition, it was also shown that an isoform that skips the third exon of LRP6 and displays reduced Wnt/β-catenin signaling activation is significantly augmented in the brains of patients with Alzheimer’s disease (Alarcón et al., 2013). When *LRP6* is specifically deleted in the forebrain, synapse formation is suppressed while amyloid-β accumulation and neuronal apoptosis are promoted, altogether resulting in aggravation of Alzheimer’s disease symptoms (Liu et al., 2014). Consistently, in Alzheimer’s disease patients, *DKK1* is highly expressed and causes suppression of LRP6-amyloid precursor protein (APP)-mediated Wnt/β-catenin signaling, which results in accumulation of amyloid-β and synapse loss (Elliott et al., 2018). These data suggest that dysregulation of LRP6 function in the brain leads to suppression of Wnt/β-catenin signaling and exacerbation of Alzheimer’s disease symptoms. A positive role of LRP6 for neuronal activity has also been reported. Through genetic screening, LRP6 was found to localize to excitatory synapses of mature neurons and promote synaptogenesis (Sharma et al., 2013), and Wnt3a and Wnt8 have been shown to cooperate with LRP6 in this process (Avila et al., 2010; Sharma et al., 2013). Moreover, APP, also known as precursor of amyloid-β, interacts with LRP6 and activates Wnt/β-catenin signaling, leading to enhanced synaptic stability (Elliott et al., 2018).

It is well-known that mutations in *PARK8* are implicated in Parkinson’s disease (Kumari and Tan, 2009). LRRK2, a product of *PARK8* gene, interacts with LRP6 and acts as a scaffold between LRP6 and the β-catenin destruction complex (Berwick and Harvey, 2012). Pathogenic mutations in LRRK2 lead to reduced interaction with LRP6, suppressing Wnt/β-catenin signaling (Berwick and Harvey, 2012).

LRP6 also plays a protective role in brain ischemic injury (Abe et al., 2013). Compared to wild-type mice, more areas of the brain are damaged through ischemic injury in LRP6^+/–^ mice. GSK3β activity and expression of inflammatory marker genes are also increased in the brains of LRP6^+/–^ mice (Abe et al., 2013).

### Metabolic Syndrome

Metabolic syndrome is characterized by abnormal levels of metabolites (e.g., sugars and lipids) in the body and is highly associated with cardiovascular disease and diabetes. Risk factors for metabolic syndrome are diet, low physical activity, aging, and genetics (Rochlani et al., 2017). The relationship between LRP6 dysfunction and metabolic syndrome has been widely studied.

It is well known that dysregulation of LRP6 is highly associated with coronary artery disease (CAD) and atherosclerosis. Through genome-wide analysis of CAD patients, R473Q, R360H, N433S, and R611C residue mutations in LRP6 were found to be correlated with CAD pathogenesis, as determined by high glucose, lipid, and low-density lipoprotein (LDL) levels in blood vessels (Mani et al., 2007; Singh et al., 2013b). In addition, the LRP6 R611C mutation, which does not effectively activate Wnt/β-catenin signaling compared to wild-type LRP6, leads to low LDL uptake and clearance. Taken together, these data suggest that LRP6 is a critical modulator of receptor-mediated LDL endocytosis (Mani et al., 2007; Liu et al., 2008; Ye et al., 2012).

Abnormal proliferation of vascular smooth muscle cells (VSMC) via activation of PDGF signaling is a well-known cause of atherosclerosis (Raines, 2004). Wild-type LRP6 interacts with PDGF receptor-β and causes its lysosomal degradation, a function that is impaired in the LRP6 R611C form (Keramati et al., 2011). As a result, VSMC proliferation through PDGF signaling is increased in Lrp6^R611C/R611C^ mutants (Keramati et al., 2011). Moreover, VSMCs from Lrp6^R611C/R611C^ mice exhibit suppressed Wnt/β-catenin signaling but increased non-canonical Wnt signaling, a shift that results in the activation of PDGF signaling via SP1 (Srivastava et al., 2015). Consequently, VSMCs are maintained in an undifferentiated form in the arterial wall, further increasing their proliferation and causing them to migrate at accelerated rates (Srivastava et al., 2015). In summary, impairment of LRP6 activity is highly correlated with CAD through PDGF signaling. Finally, the miRNA-17∼92 cluster targets LRP6 and downregulates Wnt/β-catenin signaling, and deficiency of miRNA-17∼92 in endothelial cells improves blood flow and arteriogenesis (Landskroner-Eiger et al., 2015).

The LRP6 R611C mutant form is also associated with altered insulin signaling. R611C mutation of LRP6 in skeletal muscle suppresses *TCF7L2*-dependent transcription of the insulin receptor (IR) and reduces its protein levels. This results in low insulin sensitivity and high glucose level in blood vessels, both of which contribute to type II diabetes (Singh et al., 2013a).

Additionally, Lrp6^R611C/R611C^ mice maintain a high level of LDL and lipids in the plasma, which induces fatty liver (Go et al., 2014). In Lrp6^R611C/R611C^ mutant hepatocytes, IGF/Akt/mTORC1/2 signaling and lipid synthesis are activated, and treatment with the mTOR inhibitor rapamycin or recombinant Wnt3a rescue these pathogenic effects (Go et al., 2014).

Cardiac-specific knockout of LRP6 activates dynamin-related protein 1 (Drp1) in heart tissue and reduces carnitine palmitoyltransferase 1b (CPT1b) (Wang et al., 2020). Since CPT1b is an enzyme involved in lipid oxidation, downregulation of CPT1b levels due to LRP6 deficiency causes lipid accumulation in heart tissue and reduces left ventricular ejection fraction (LVEF), altogether leading to cardiac dysfunction (Wang et al., 2020).

### Inflammation

Organ homeostasis is maintained through the coordinated action of inflammatory cytokines with host defense function, and dysregulation of inflammatory cytokines is implicated in immune disease or cancer (Greten and Grivennikov, 2019). Moreover, inflammatory cytokines can regulate Wnt/LRP6 signaling. For instance, long exposure to pro-inflammatory cytokine interferon-γ or TNF-α induces Dkk1 expression and inhibits Wnt/β-catenin signaling, leading to increased intestinal inflammation (Nava et al., 2010). Ileal Crohn’s disease (CD) is a disease that causes pain, diarrhea, and malnutrition due to chronic inflammation in the intestine (Koslowski et al., 2012). Genome-wide analysis from CD patients revealed an association between the Ile1062Val LRP6 SNP with early disease onset. Lower levels of LRP6 mRNA were also detected in these patient samples (Koslowski et al., 2012). Dendritic cell (DC)-specific knockout of LRP5/6 promotes differentiation of effector T cells and represses regulatory T cell differentiation, resulting in enhanced anti-tumor immunity and inhibition of tumor growth, both of which show that fine regulation of LRP6 is essential for proper immune responses (Hong et al., 2016).

### Skeletal Disease

Bone mass formation and maintenance is regulated by the activity of osteoblasts, which form bone, and osteoclasts, which degrade bone. Dysregulation of bone mass leads to osteoporosis or sclerosteosis, diseases that are heavily influenced by genetic factors (Regard et al., 2012). For instance, LRP5 mutations generally lead to decreased bone mass and osteoporosis due to reduced Wnt/β-catenin signaling (Gong et al., 2001). One exception is the G171V mutation in LRP5, which increases rather than decreases bone mass (Babij et al., 2003). LRP6 is a paralog of LRP5, and studies on the association between LRP6 and bone homeostasis have also been performed. For example, heterozygous deficiency of LRP6 in mice leads to a reduction in bone mineral density (BMD) (Holmen et al., 2004). Moreover, combination of LRP6 heterozygous deficiency with LRP5 homozygous deficiency, leads to a greater reduction in BMD compared to LRP5 homozygous deficiency alone (Holmen et al., 2004). Tissue-specific ablation of LRP5 and LRP6 in the mesenchyme, which is the precursor of skeletal tissue, leads to embryonic skeletal defects, similar to the phenotype seen upon mesenchyme-specific deletion of β-catenin (Joeng et al., 2011).

Several proteins that bind to LRP6 regulate bone formation via modulation of Wnt/β-catenin signaling. It is well-known that loss-of-functions mutation in sclerostin (expressed by the *SOST* gene) cause sclerosteosis (Balemans et al., 2001). Sclerostin inhibits Wnt/β-catenin signaling by binding to LRP6 and disrupting FZD-LRP6 interaction (Li et al., 2005; Semënov et al., 2005). Biglycan, a type of proteoglycan, activates Wnt/β-catenin signaling by maintaining the interaction between Wnt3a and LRP6, and deficiency of biglycan compromises bone formation (Berendsen et al., 2011).

Parathyroid hormone interacts with PTH1R to promote LRP6 phosphorylation and activate Wnt/β-catenin signaling in osteoblasts, leading to increased bone formation (Wan et al., 2008). Osteoblast-specific knockout of *LRP6* reduces the expression of osteoblast differentiation-related genes and suppresses bone formation, even in the presence of PTH (Li et al., 2013, 2016).

Oxidized phospholipids bind to LRP6 and reduce LRP6 plasma membrane levels via clathrin-dependent endocytosis (Wang et al., 2018). As a result, phosphorylation of LRP6 and Wnt/β-catenin signaling are reduced, inhibiting osteoblast differentiation (Wang et al., 2018). It has also been shown that oxidized phospholipid levels are high in patients suffering from hyperlipidemia, suggesting that LRP6 may have an important role in the occurrence of osteoporosis via hyperlipidemia (Wang et al., 2018).

## Perspectives (Closing Remarks)

It has been more than 20 years since LRP6 was discovered as a co-receptor for Wnt/β-catenin signaling. Most studies focusing on the mechanisms underlying LRP6-mediated Wnt/β-catenin signaling have concentrated on phosphorylation events in the intracellular domain of LRP6. However, recent reports have determined that additional PTMs such as ubiquitination, acetylation, and *O*-GlcNAcylation are also essential for regulating LRP6 activity. Moreover, LRP6 is involved in multiple signaling cascades apart from Wnt/β-catenin. These include, but are not limited to, non-canonical Wnt signaling, GPCR signaling, cell cycle-related signaling, and Hippo signaling (Figure 5). Until now, the occurrence of disease via dysregulation of LRP6 has been primarily linked to aberrant Wnt/β-catenin signaling. However, we suggest that future studies focusing on LRP6 and disease should also investigate the involvement of other signaling pathways. Moreover, since single point mutations in LRP6 are linked to multiple diseases, it would be worthwhile to analyze the cross-talk between these diseases, and to more thoroughly decipher their connections to LRP6. Considering the complex mechanisms surrounding the regulation and activation of LRP6, as well as its important role in disease occurrence, LRP6 is thus expected to be an attractive therapeutic target in future studies.

## Author Contributions

WJ planned and wrote the manuscript. EJ directed, edited, and finalized the manuscript. Both authors read and approved the final version of the manuscript.

## Conflict of Interest

The authors declare that the research was conducted in the absence of any commercial or financial relationships that could be construed as a potential conflict of interest.

## Publisher’s Note

All claims expressed in this article are solely those of the authors and do not necessarily represent those of their affiliated organizations, or those of the publisher, the editors and the reviewers. Any product that may be evaluated in this article, or claim that may be made by its manufacturer, is not guaranteed or endorsed by the publisher.
